# Dual-phase whole-heart imaging using image navigation in congenital heart disease

**DOI:** 10.1186/s12880-018-0278-0

**Published:** 2018-10-16

**Authors:** Danielle M. Moyé, Tarique Hussain, Rene M. Botnar, Animesh Tandon, Gerald F. Greil, Adrian K. Dyer, Markus Henningsson

**Affiliations:** 1 Department of Pediatrics, Division of Cardiology, UT Southwestern Medical Center Dallas, Dallas, TX USA; 2 Department of Pediatrics, Division of Cardiology, Children’s Health, Children’s Medical Center Dallas, Dallas, TX USA; 3 Departments of Radiology and Biomedical Engineering, University of Texas Southwestern Medical Center, Dallas, TX USA; 4 Division of Imaging Sciences, King’s College London, London, UK; 5 Pontificia Universidad Católica de Chile, Escuela de Ingeniería, Santiago, Chile; 6 Pediatric Cardiology, Children’s Health Children’s Medical Center of Dallas, 1935 Medical District Drive, Dallas, TX 75235 USA

**Keywords:** Steady-state free precession MRI, Congenital heart disease, Dual phase imaging, Respiratory motion correction

## Abstract

**Background:**

Dual-phase 3-dimensional whole-heart acquisition allows simultaneous imaging during systole and diastole. Respiratory navigator gating and tracking of the diaphragm is used with limited accuracy. Prolonged scan time is common, and navigation often fails in patients with erratic breathing. Image-navigation (iNAV) tracks movement of the heart itself and is feasible in single phase whole heart imaging. To evaluate its diagnostic ability in congenital heart disease, we sought to apply iNAV to dual-phase sequencing.

**Methods:**

Healthy volunteers and patients with congenital heart disease underwent dual-phase imaging using the conventional diaphragmatic-navigation (dNAV) and iNAV. Acquisition time was recorded and image quality assessed. Sharpness and length of the right coronary (RCA), left anterior descending (LAD), and circumflex (LCx) arteries were measured in both cardiac phases for both approaches. Qualitative and quantitative analyses were performed in a blinded and randomized fashion.

**Results:**

In volunteers, there was no significant difference in vessel sharpness between approaches (*p* > 0.05). In patients, analysis showed equal vessel sharpness for LAD and RCA (*p* > 0.05). LCx sharpness was greater with dNAV (*p* < 0.05). Visualized length with iNAV was 0.5 ± 0.4 cm greater than that with dNAV for LCx in diastole (p < 0.05), 1.0 ± 0.3 cm greater than dNAV for LAD in diastole (*p* < 0.05), and 0.8 ± 0.7 cm greater than dNAV for RCA in systole (*p* < 0.05). Qualitative scores were similar between modalities (*p* = 0.71). Mean iNAV scan time was 5:18 ± 2:12 min shorter than mean dNAV scan time in volunteers (*p* = 0.0001) and 3:16 ± 1:12 min shorter in patients (*p* = 0.0001).

**Conclusions:**

Image quality of iNAV and dNAV was similar with better distal vessel visualization with iNAV. iNAV acquisition time was significantly shorter. Complete cardiac diagnosis was achieved. Shortened acquisition time will improve clinical applicability and patient comfort.

## Background

Three-dimensional (3D) whole-heart imaging for assessment of morphology in congenital heart disease has revolutionized how images have been obtained in the last decade [1, 2]. Sørensen et al. [3] evaluated the diagnostic ability and utility of 3D whole heart balanced steady-state free precession (3D WH bSSFP) MRI for morphology in congenital heart disease with single-phase imaging. All images were acquired in end diastole and scanning was performed during free breathing with a pencil beam navigator (dNAV) on the right hemi-diaphragm. They showed the 3D WH bSSFP to be reliable and accurate in the assessment of morphology in congenital heart disease. This showed that 3D, single phase imaging with the conventional navigational technique (dNAV) was superior to the two-dimensional (2D) scanning that was being done at that time. Uribe et al. [4] extended this analysis to dual phase imaging with dNAV in an attempt to determine optimal coronary artery imaging timing. They found that optimal timing (systolic versus diastolic rest period) was patient dependent and different for each coronary artery segment, favoring dual phase whole heart imaging to allow for optimal coronary artery visualization by retrospectively selecting the optimal imaging rest period. Furthermore, in previous work by Hussain et al. [5], it was shown that images obtained during the systolic rest period offer better clarity for many cardiac segments in congenital heart disease, including all four cardiac chambers and pulmonary veins in particular. Diastolic imaging, however, was preferable when imaging the aorta and branch pulmonary arteries. This finding of some cardiac structures imaging better during the systolic rest period while other structures image better during the diastolic rest period led to the thought that dual phase imaging would be superior in diagnostic quality when compared with either single phase alone, which they illustrated.

Technically, dual-phase 3D WH bSSFP is an extension of conventional 3D WH bSSFP, which is acquired in either systole or diastole, and uses T2 preparation [6] and fat suppression pre-pulses to improve contrast. To compensate for respiratory motion, a one-dimensional navigator is typically used, positioned over the right hemi-diaphragm and measures respiratory motion in the foot-head (FH) direction [7]. This pencil beam diaphragmatic navigator signal is used to gate the 3D WH bSSFP scan to end-expiration using a narrow gating window, typically 5 mm, resulting in a scan efficiency of approximately 30–50%. Additionally, the dNAV can be used to correct for the translational respiratory FH motion of the heart using an estimated correction factor to convert translational motion of the diaphragm to that of the heart. For dual-phase 3D WH bSSFP, a dNAV acquisition is performed for both the systolic and diastolic rest phases where each dNAV has a separate 5 mm gating window. Systolic and diastolic 3D WH bSSFP data for any given cardiac cycle, however, are only accepted if both the systolic and diastolic dNAV are within their individual gating windows [8]. This further lowers the scan efficiency, extending the total dual-phase 3D WH bSSFP scan time. Furthermore, although dNAV can adequately suppress respiratory motion artifact for 3D WH bSSFP in most patients, for some patients respiratory artifacts remain, leading to lower diagnostic yield [9]. Additionally, the dNAV navigator often fails in cases of erratic breathing patterns, resulting in the inability to acquire necessary images.

In recent years, image-based navigation (iNAV) has been proposed for single-phase whole heart bSSFP to enable direct measurement and respiratory motion correction of the heart itself rather than the diaphragm [10–13]. With this approach, 2D or 3D real-time images are acquired, which allow localization of the heart and differentiation from surrounding structures. One approach to iNAV acquisition involves adding phase encoding gradients to the start-up echoes of a bSSFP sequence to generate a 2D image. Although this results in low spatial resolution in the phase encoding direction and projection of the field-of-view (FOV) in the slice encoding direction, it is sufficient to track the respiratory motion of the heart provided that the acquisition is oriented with readout along FH direction (high spatial resolution). In addition to correcting for translational respiratory motion of the heart, gating has been implemented for iNAV, using either respiratory bellows [14] or more recently the Constant Respiratory efficiency UsIng Single End-expiratory threshold (CRUISE) technique [15]. The CRUISE algorithm predefines a 50% gating efficiency whereby k-space is fully acquired during the first half of the 3D WH bSSFP scan and the most motion corrupted data are discarded and re-acquired in the second half. A challenge of extending iNAV to dual-phase 3D WH bSSFP is the different cardiac motion states between systole and diastole, which precludes the use of a common navigator for both phases, as cardiac motion may be interpreted as respiratory motion. This issue can be addressed by using separate iNAV reference frames for systole and diastole. Further, the use of separate systolic and diastolic iNAVs using CRUISE gating may improve dual-phase 3D WH bSSFP gating efficiency as the CRUISE algorithm does not require both systolic and diastolic navigators to be within a pre-defined gating window for a given cardiac cycle.

The purpose of this study was to implement and evaluate the diagnostic ability of a new iNAV approach for dual-phase 3D WH bSSFP in healthy volunteers and patients with congenital heart disease, using separate systolic and diastolic image navigators with CRUISE gating.

## Methods

Approval was obtained from the University of Texas Southwestern Institutional Review board and the local ethics committee before initiation of the study. All participants provided written informed consent and all data were collected per standard of care at Children’s Health Children’s Medical Center Dallas and the University of Texas Southwestern Medical Center. All experiments were performed on a 1.5 T clinical scanner (Ingenia, Philips Healthcare, Best, The Netherlands) using a 32-channel cardiac coil.

### Dual-phase 3D WH bSSFP with iNAV motion compensation

**Fig. 1:**
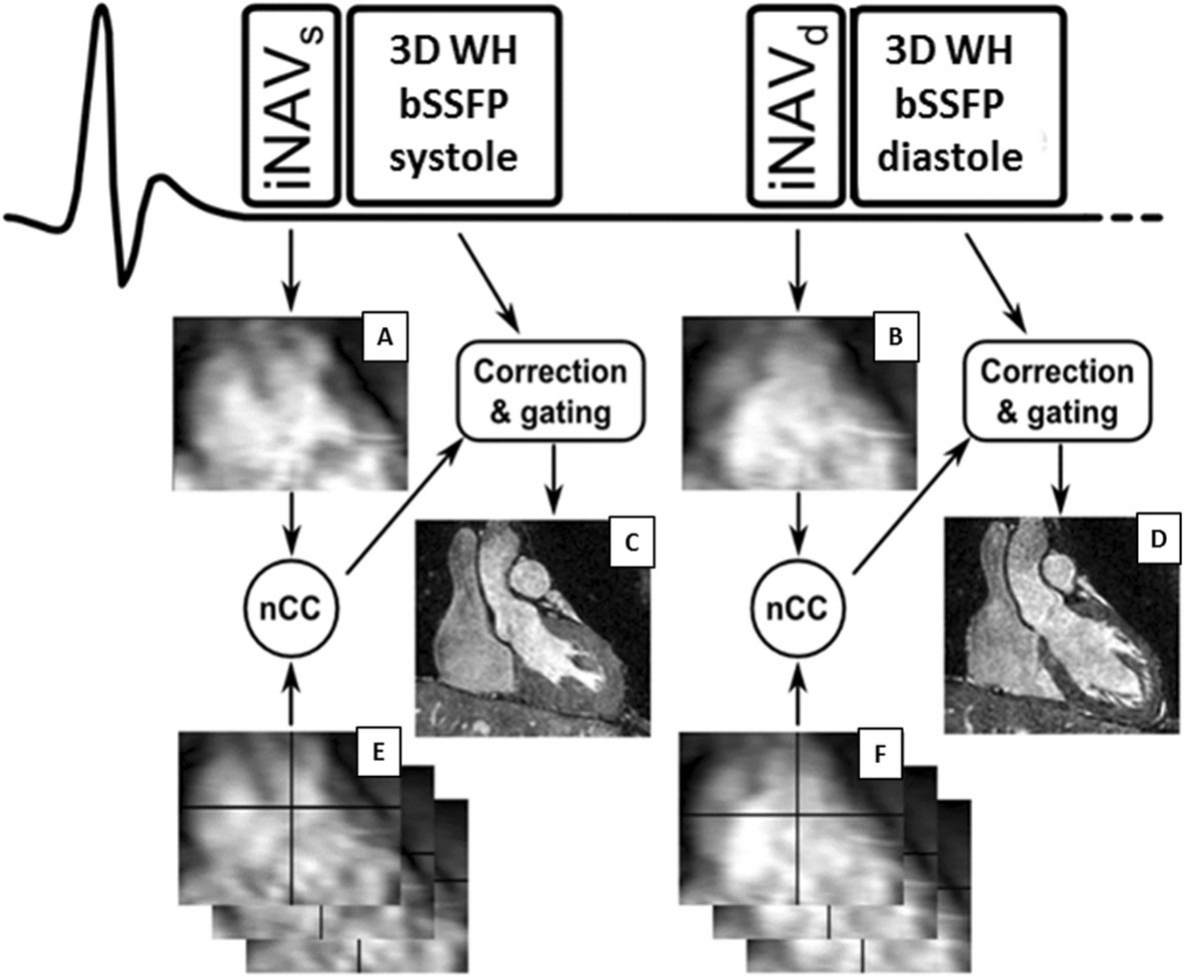
This describes the schematics of iNAV respiratory motion correction when applied to dual-phase 3D WH bSSFP. A separate iNAV reference (iNAV REF) was used for systole (**a**) and diastole (**b**). All subsequent systolic and diastolic iNAVs (**e** and **f**) were registered to their respective reference iNAV (**a** and **b** respectively) using normalized cross-correlation (nCC). Images **c** and **d** show the reconstructed images obtained

**Fig. 2:**
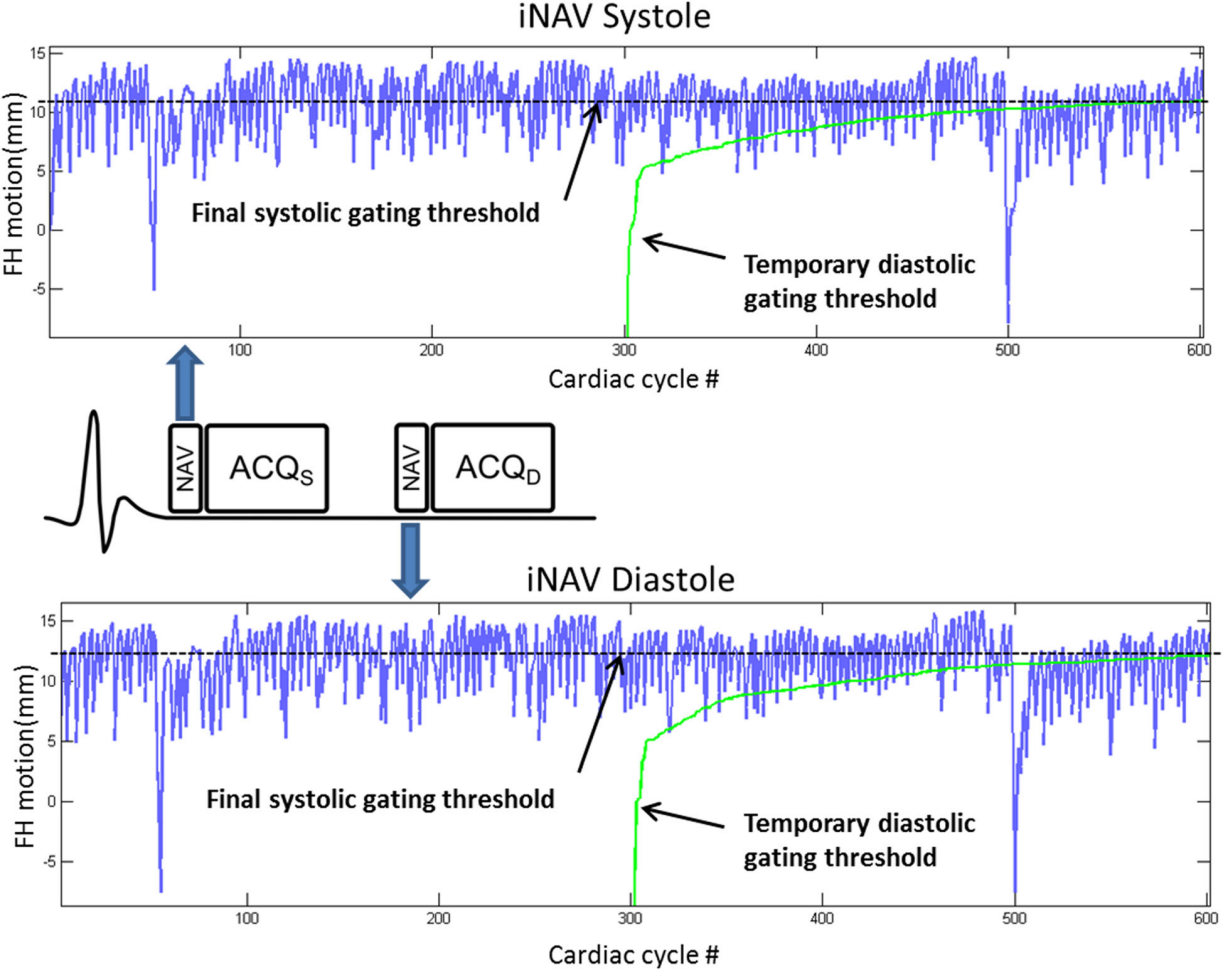
This describes the schematic for the application of CRUISE gating to Dual-phase 3D WH bSSFP with separate systolic and diastolic navigators. FH: Foot-head; ACQ_S_: systolic acquisition; ACQ_D_: diastolic acquisition Top panel: image navigator in systole. Bottom panel: image navigator in diastole. The oscillating line (blue line) represents the calculated image position compared to end-expiration (top line of graph). 600 cardiac cycles are used to generate the final gating threshold. In the first 300 cardiac cycles 3D WH bSSFP k-space was completely filled at any respiratory position. The temporary gating threshold is created by this initial data (green line), which represents the worst (most inspiratory) navigator position. During the second 300 cardiac cycles, the most motion corrupted 3D WH bSSFP k-space segment, as defined by the most inspiratory navigator position, was discarded and re-measured in the following cardiac cycle. If the FH position of the re-acquired segment was higher (closer to expiration) than the temporary inspiratory gating thresh-hold, it was kept, resulting in an updated gating thresh-hold. On the other hand, if the position of the re-acquired segment fell below the threshold it was ignored and re-acquired in the subsequent cardiac cycle. The temporary gating threshold improves until a final gating threshold is reached

The proposed iNAV motion correction strategy for dual-phase 3D WH bSSFP is shown in Fig. 1. The iNAVs were acquired by adding phase encoding gradients to 10 startup echoes of the bSSFP pulse sequence as previously described [10]. To account for differences in each cardiac motion state, separate iNAV references were acquired for the systolic and diastolic rest phase. The iNAV references were defined as the first acquired iNAV in each cardiac phase. Each subsequent iNAV for a given phase was registered to its corresponding iNAV reference using normalized cross-correlation. Translational motion correction was performed in FH and left-right (LR) direction. Additionally, respiratory gating was implemented using the CRUISE algorithm, where a predefined gating efficiency of 50% was used. This gating efficiency has been shown to have an equivalent dNAV gating window of 5 mm and had a similar, if not slightly superior efficiency. The details of this CRUISE algorithm have recently been described [15]. For the dual-phase implementation of CRUISE, the systolic and diastolic iNAVs were separately gated to end-expiration, as shown in Fig. 2. The iNAV motion correction and gating were implemented in real-time on the scanner and no post-processing of the 3D WH bSSFP was required.

### MRI experiments

All subjects underwent dual-phase 3D WH bSSFP with dNAV and independent systolic and diastolic iNAV correction and CRUISE gating. For comparison, a dual-phase scan was acquired using dNAV with a 0.6 tracking factor and a 5 mm gating window. As previously outlined, the dual phase implementation of dNAV gating required both systolic and diastolic navigators to be within the 5 mm gating window for the k-space segment of a particular cardiac cycle to be accepted. The 3D WH bSSFP sequence had the following image parameters: FOV = 300 × 300 × 100 mm (coronal orientation), Δx = 1.5 × 1.5 × 1.5 mm^3^, α = 70°, echo time 1.7 ms, repetition time 3.4 ms, SENSE factor = 2.5 (phase encoding direction). Fat suppression and T2-preparation pulses were used to improve contrast (T2 prep time = 50 ms) [16]. ECG-triggering using subject-specific delay times were determined using high temporal resolution cine 4 chamber scans to coincide with the systolic and diastolic rest periods. The systolic and diastolic data acquisition windows were identical and defined as the shortest rest period of the two cardiac phases, typically approximately 90 msec. With these imaging parameters and a heart-rate of 70 beats-per-minute the nominal scan time was 3 min and 26 s (i.e. assuming 100% navigator efficiency). Acquisition time was recorded for each sequence.

### Healthy volunteers

Nineteen healthy subjects participated in the study and underwent dual-phase 3D WH bSSFP with both dNAV and iNAV motion compensation. The scans were acquired in a randomized order. Interim analysis of the volunteer data was performed to evaluate for potential benefit prior to starting patient recruitment.

### Patients

Thirty patients undergoing a non-sedated clinically indicated cardiac MRI for congenital or pediatric heart disease were included in the study. The dual phase 3D WH bSSFP scans with both dNAV and iNAV motion compensation were acquired in a randomized order at the end of each patient’s clinically indicated study. Gadobutrol contrast was used in 28/30 (93%) patients and contrast agent was administered as part of the clinically indicated study, prior to study image acquisition. For all patients receiving gadolinium, the first whole-heart sequence was started from 4 to 8 min post dose. No patient, by study design, was scanned under anesthesia. Patient demographics and clinical indications for cardiac MRI are shown in Table 1.

**Table 1:** Patient Demographics and Clinical Indications for cardiac MRI

Age, (years, mean ± standard deviation):	16.7 ± 5.1
Male gender, N (%)	13 (43%)
Ethnicity, N (%)	
• Caucasian	22 (73%)
• Hispanic	5 (17%)
• Asian	2 (7%)
• African American	1 (3%)
Clinical indications for cardiac MRI, N (%)	
• Congenital heart disease post-surgical repair	16 (53%)
Underlying cardiac disease:	
○ Tetralogy of Fallot	
○ Tetralogy of Fallot, absent pulmonary valve	
○ Total anomalous pulmonary venous return	
○ DORV, D-TGA, VSD, pulmonary stenosis	
○ DORV, D-TGA, VSD, aortic stenosis	
○ Coarctation of the aorta	
○ Coarctation of the aorta, VSD, bicuspid aortic valve	
○ Turner syndrome, partial anomalous pulmonary venous return	
○ Unbalanced AVCD, DORV, L-TGA, pulmonary stenosis	
○ AVCD, pulmonary stenosis, pulmonary regurgitation	
○ Ebstein’s malformation of the tricuspid valve	
○ Pulmonary atresia, intact ventricular septum	
○ Pulmonary valve regurgitation	
○ Pulmonary stenosis, sinus venosus defect	
○ Shone’s complex	
○ Partial anomalous pulmonary venous return, Atrial septal defect	
• Hypertrophic cardiomyopathy	4 (13%)
• Evaluation for arrhythmogenic right ventricular cardiomyopathy/dysplasia	2 (7%
• Bicuspid aortic valve; evaluation of aortic dilation	3 (10%)
• Loeys-Dietz syndrome	1 (3%)
• Evaluation for myocarditis	1 (3%)
• Suspected abnormal left coronary artery origin	1 (3%)
• Kawasaki disease with giant aneurysms	1 (3%)
• Ectopic atrial tachycardia; evaluate cardiac anatomy	1 (3%)

*DORV* Double outlet right ventricle, *TGA* transposition of the great arteries, *VSD* ventricular septal defect, *AVCD* Atrioventricular canal defect

### Data and statistical analysis

The dual phase 3D WH bSSFP datasets were reformatted using dedicated software (Soap-bubble software tool [Release 5.1 for PRIDE, Philips Healthcare, Best the Netherlands]) [17] to visualize the right coronary artery (RCA), left anterior descending (LAD) artery and left circumflex (LCx) artery. Vessel sharpness and visualized length were measured for the RCA, LAD and LCx for both cardiac rest phases and for both motion correction navigation methods according to previously validated techniques [4, 14]. All variables were tested for normality of distribution using a one-sample Kolmogorov-Smirnov Test. Descriptive statistics such as mean and standard deviation were used for normally distributed variables. Median and range are used for variables with a non-parametric distribution and variables are compared using a Wilcoxon-signed rank test. The 95% confidence intervals of normally distributed measurements were reported and a paired t-test was employed to assess the difference between them. All statistical analyses were performed using SAS 9.4 (SAS Institute, Cary, NC). Qualitative analysis was performed using a previously described and validated visual score [7, 10] on reformatted images of each coronary artery in a blinded and randomized fashion by two readers (AT and AD, with three and 5 years of experience in cardiovascular MR imaging respectively). The scoring was performed using a consensus agreement as previously described [5]. Additionally, each cardiac or great vessel structure was scored as to whether its connection was visualized with diagnostic adequacy. Adequate visualization was defined by the structure being adequately defined without severe blurring (i.e. score ≥ 3 out of 5, using a previously described scoring system [18]). A generalized linear model was used to determine the effect on coronary artery, systolic or diastolic phase and navigator type on image quality score.

## Results

### Healthy volunteers

**Fig. 3:**
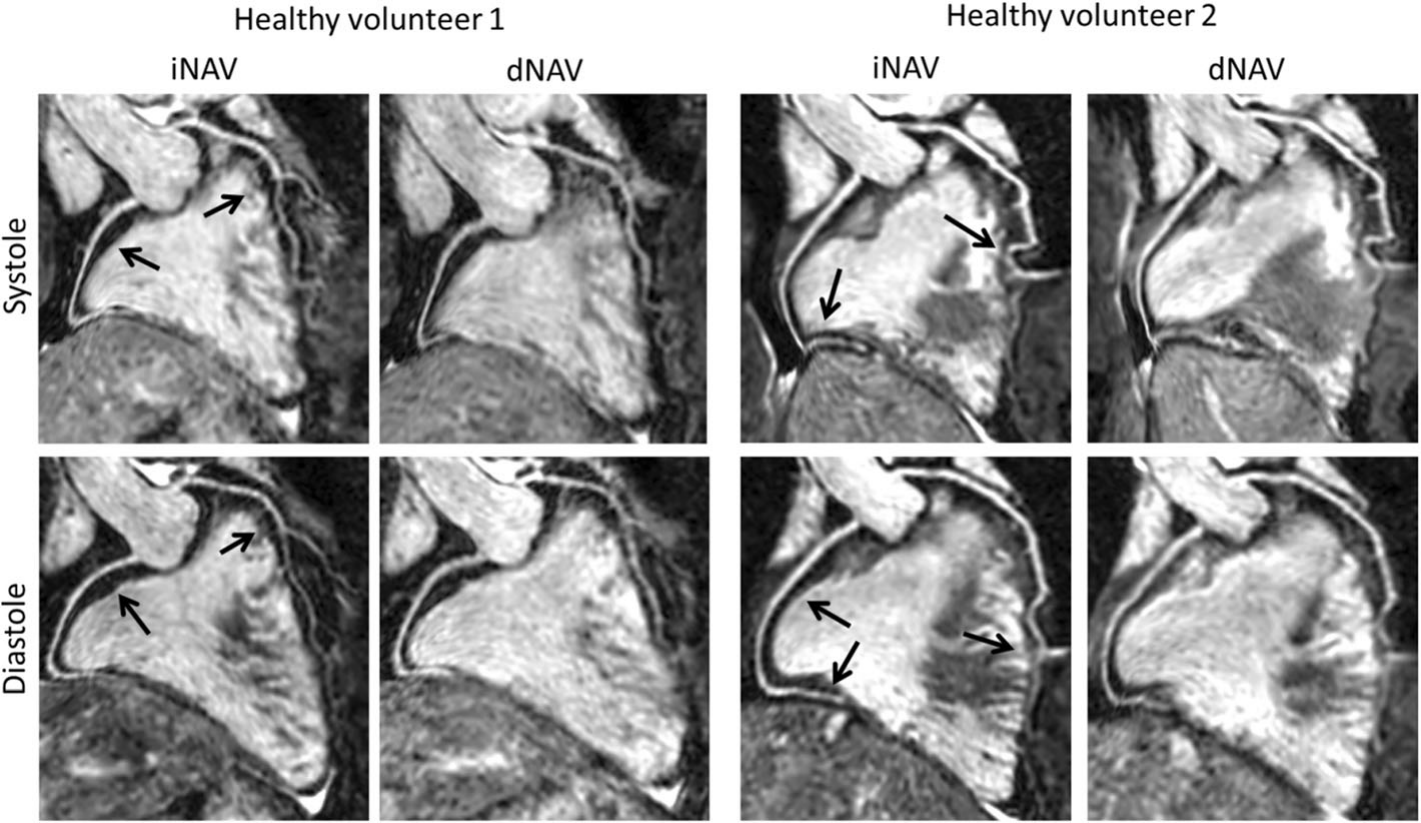
Reformatted dual-phase 3D WH bSSFP data from two healthy subjects in systole and diastole. This figure shows that in some subjects with poor dNav gating, excessively long scan times can result in worse image quality. iNAV = image-navigator; dNAV = diaphragmatic 1D pencil beam navigator. Arrows highlight coronary segments with improved sharpness using iNAV compared to dNAV

Dual phase 3D WH bSSFP was successfully obtained in all nineteen volunteers (7 male, age (mean ± standard deviation): 33.1 ± 8.4 years) using iNAV and dNAV motion compensation. The mean scan time using iNAV was 5:33 ± 1:50 min (min), was significantly shorter than that of dNAV which was 9:51 ± 4:02 min (*p* = 0.0001). In 17 out of 19 volunteers, the scan time was longer using dNAV and in one case, the dNAV method resulted in excessively long scan duration of more than 22 min. In contrast, the longest observed scan time using iNAV was 9:30 min. Reformatted dual phase 3D WH bSSFP images using iNAV and dNAV in both systole and diastole from two volunteers are shown in Fig. 3.

**Fig. 4:**
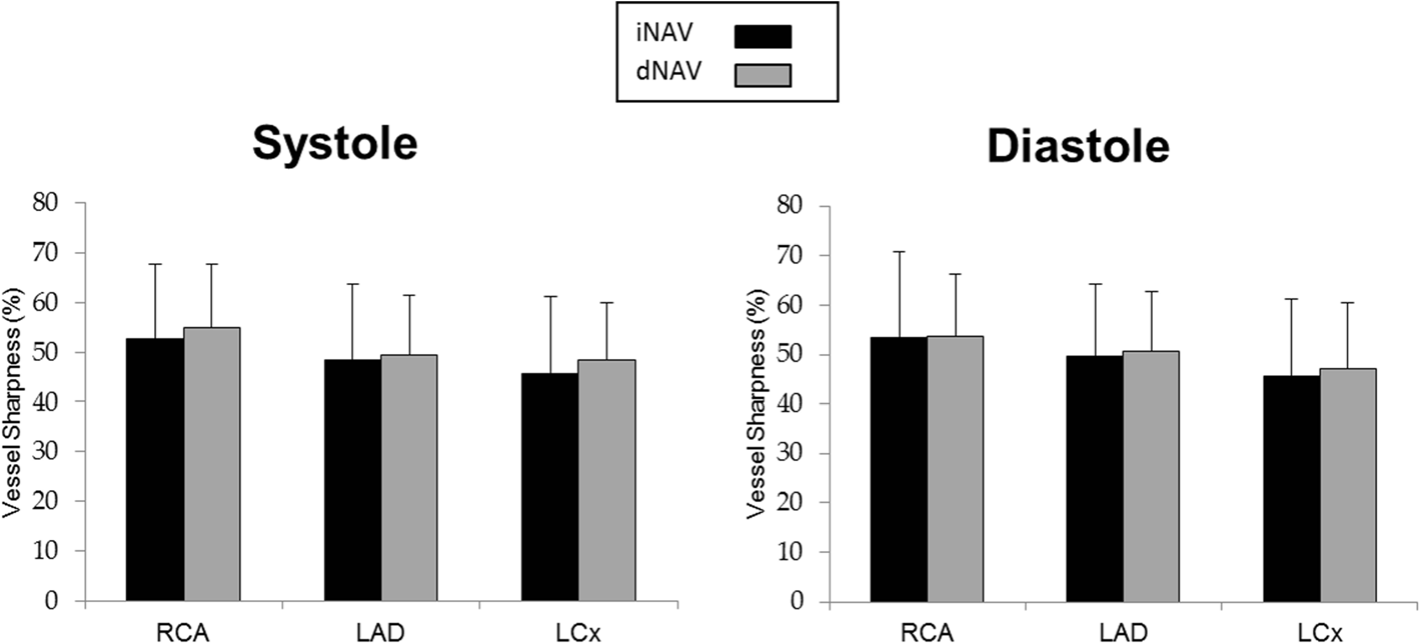
Coronary Vessel Sharpness in Volunteers. Graphical depiction of average coronary vessel sharpness for all 19 healthy subjects for systolic and diastolic 3D WH bSSFP using iNAV (black bars) and dNAV (grey bars) for motion correction. This shows equivalent sharpness for the two sequences. iNAV = image-navigator; dNAV = diaphragmatic 1D pencil beam navigator; RCA = right coronary artery; LAD = left anterior descending artery; LCx = circumflex artery

The vessel sharpness for systole and diastole of the RCA, LAD and LCx using iNAV and dNAV, averaged across all 19 healthy subjects are shown in Fig. 4. There was no statistically significant difference in vessel sharpness between images obtained with iNAV compared to those obtained with dNAV for either cardiac phase. The iNAV RCA sharpness (mean ± standard deviation) during systole was 52.69% ± 15.10% versus 54.79% ± 12.99% with dNAV (*p* = 0.17). The iNAV RCA sharpness during diastole was 53.45 ± 17.37% with iNAV 53.69% ± 12.67% with dNAV (*p* = 0.92). The LAD sharpness (mean ± standard deviation) was 48.28% ± 15.36% in systole with iNAV versus 49.49% ± 11.83% with dNAV (*p* = 0.39) and 49.55% ± 14.80% in diastole with iNAV versus 50.57% ± 12.27% with dNAV (*p* = 0.52). The LCx sharpness (mean ± standard deviation) during systole was 45.69% ± 15.56% with iNAV versus 48.42% ± 11.40% with dNAV (*p* = 0.23) and 45.56% ± 15.69% during diastole with iNAV versus 47.25% ± 13.23% with dNAV (*p* = 0.25).

### Patients

**Fig. 5:**
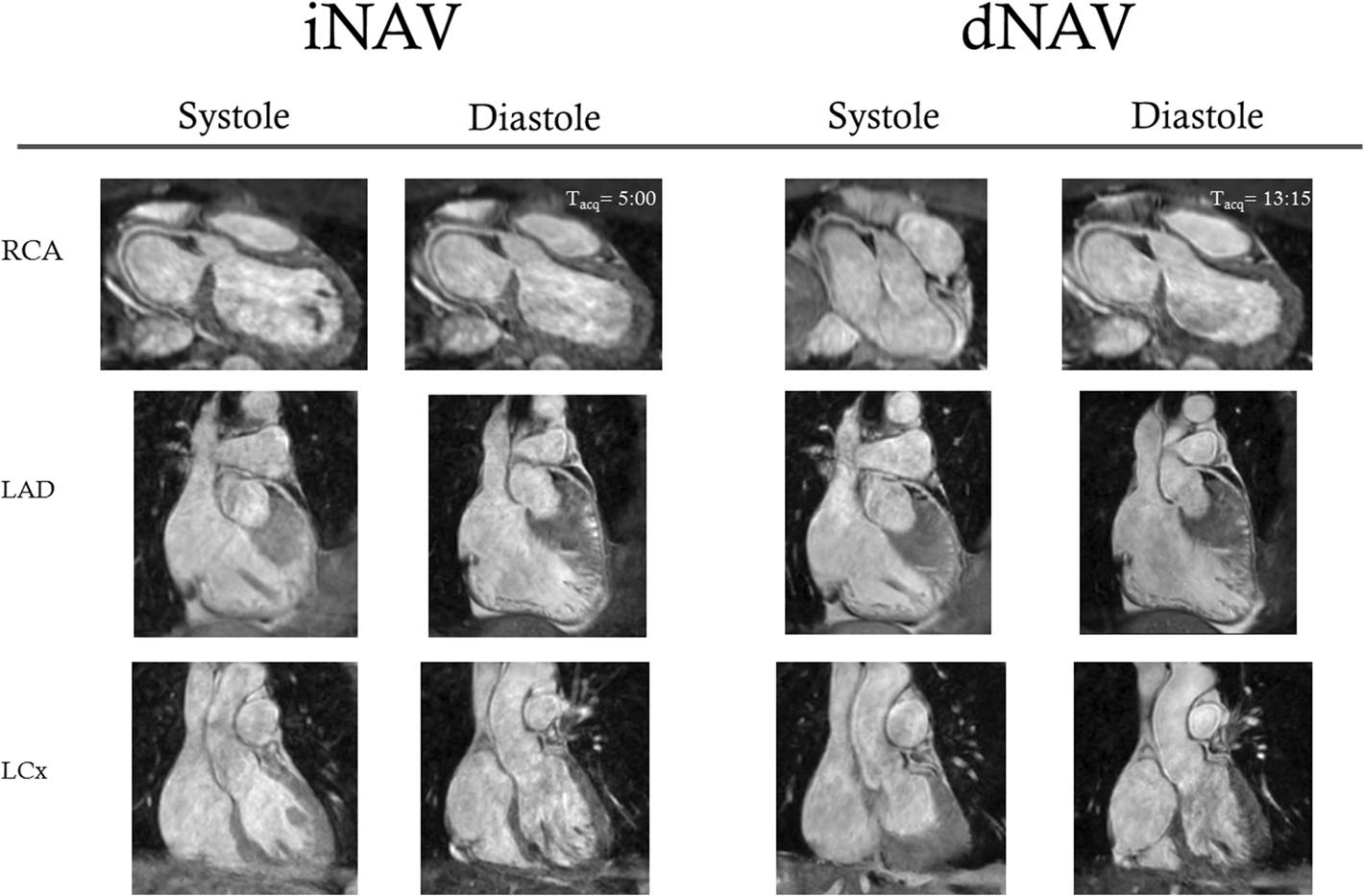
This figure shows representative reformatted dual-phase 3D WH bSSFP data from one patient obtained with both techniques using “Soapbubble” reformatting [17]. These were the images used for consensus scoring. iNAV = image-navigator; dNAV = diaphragmatic 1D pencil beam navigator; T_acq_ = acquisition time RCA = right coronary artery; LAD = left anterior descending artery; LCx = circumflex artery

Thirty-two patients underwent dual phase 3D WH bSSFP imaging. Two patients failed dNAV imaging due to erratic breathing patterns resulting in extremely low gating efficiency, and were excluded from analysis. Representative pictures of reformatted dual phase 3D WH bSSFP images using iNAV and dNAV in both systole and diastole from one patient are shown in Fig. 5.

**Fig. 6:**
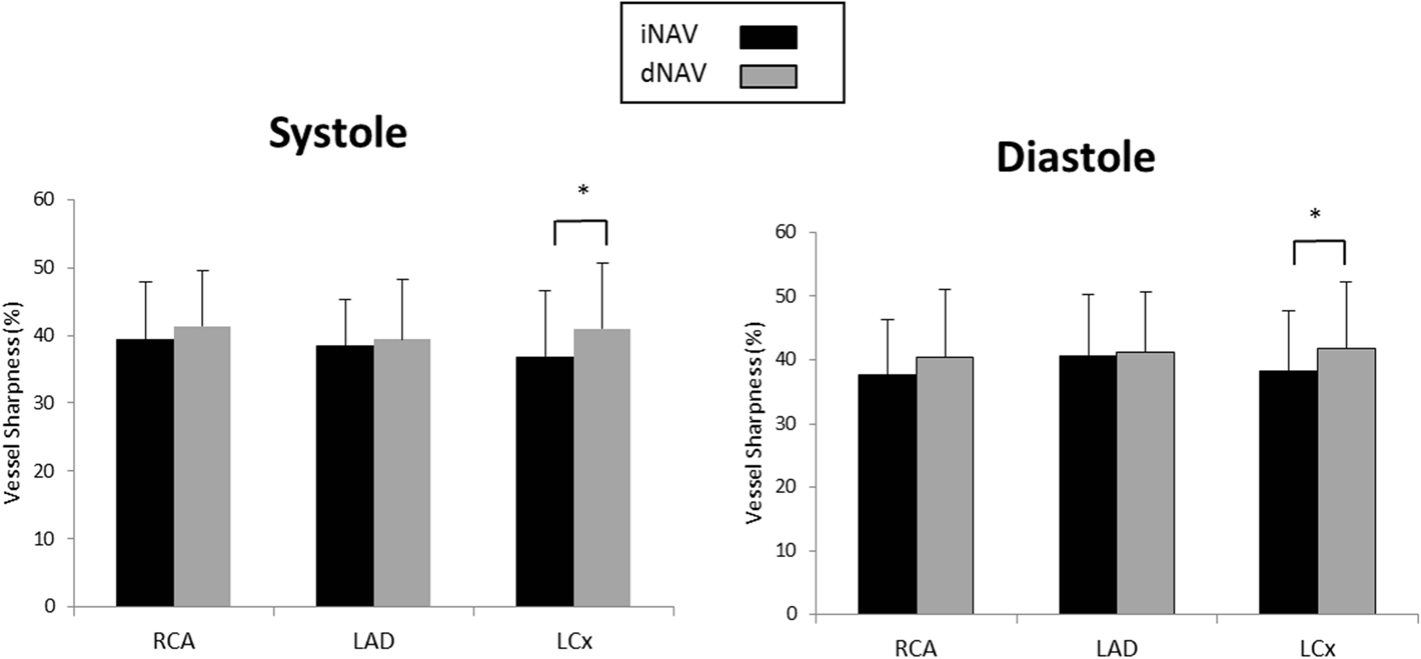
Coronary Vessel Sharpness in Patients. Graphical depiction of mean and standard deviation for coronary vessel sharpness for all 30 patients for systolic and diastolic 3D WH bSSFP using iNAV (black bars) and dNAV (grey bars) for motion correction. Statistical significance (*p* < 0.05) is signified by *. This shows largely equivalent sharpness for both phases for the two sequences with the only exception of the left circumflex in diastole. iNAV = image navigator; dNAV = diaphragmatic 1D pencil beam navigator; RCA = right coronary artery; LAD = left anterior descending artery; LCx = circumflex artery

**Fig. 7:**
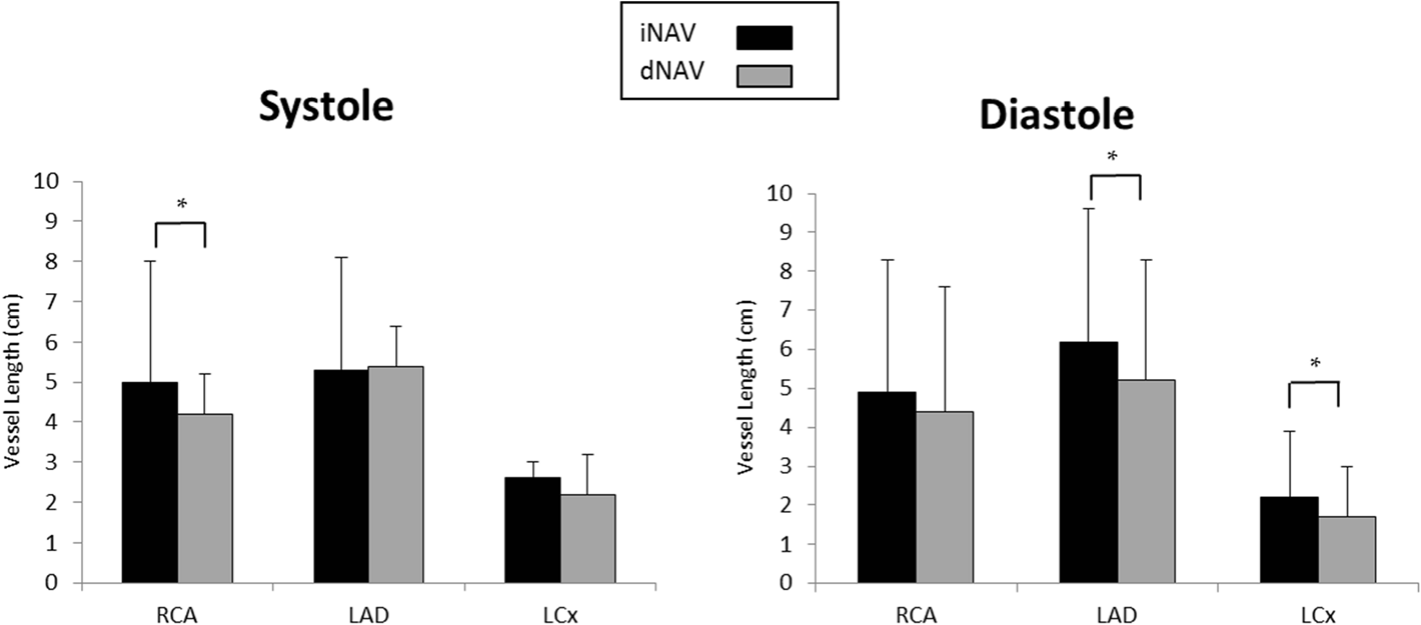
Coronary Vessel Length in Patients. Graphical depiction of mean and standard deviation for coronary vessel length for all 30 patients for systolic and diastolic 3D WH bSSFP using iNAV (black bars) and dNAV (grey bars) for motion correction. Statistical significance (p < 0.05) is signified by *. Overall, it is shown that there is a tendency for longer vessel length visualization using iNav. iNAV = image navigator; dNAV = diaphragmatic 1D pencil beam navigator; RCA = right coronary artery; LAD = left anterior descending artery; LCx = circumflex artery

Vessel sharpness was similar for the LAD and RCA in both systole (LAD mean sharpness of 38.4% ± 7.0% with iNAV versus 39.4% ± 8.9% with dNAV, *p* = 0.51; RCA mean sharpness of 39.4% ± 8.5% with iNAV versus 41.4% ± 8.2% with dNAV, *p* = 0.11) and diastole (LAD mean sharpness of 40.5% ± 9.8% with iNAV versus 41.1% ± 9.5% with dNAV, *p* = 0.61; RCA mean sharpness of 37.6% ± 8.7% with iNAV versus 40.3% ± 10.7% with dNAV, *p* = 0.13) between the two modalities. Vessel sharpness of the LCx was statistically higher with dNAV for both systole (iNAV mean sharpness of 36.8% ± 9.8% versus 41.0% ± 9.6% with dNAV, *p* = 0.007) and diastole (iNAV mean sharpness of 38.3% ± 9.5% versus 41.7% ± 10.5% with dNAV, *p* = 0.04) (Fig. 6). Visualized length, however, was significantly greater with iNAV for LCx in diastole (mean length of 2.2 ± 1.7 mm with iNAV versus 1.7 ± 1.3 mm with dNAV; *p* = 0.04), LAD in diastole (6.2 ± 3.4 mm with iNAV versus 5.2 ± 3.1 mm with dNAV; *p* = 0.02) and RCA in systole (5.0 ± 3.0 mm with iNAV versus 4.2 ± 2.3 mm with dNAV; *p* = 0.01) (Fig. 7).

There was no statistically significant effect on qualitative image score according to the type of respiratory motion compensation (PB versus iNav. OR = 0.85, 95%CI 0.60, 1.20; *p* = 0.36 phase (systole or diastole) or according to phase imaged (systole vs diastole, OR = 0.94, 95% CI 0.66, 1.32; *p* = 0.71).

**Fig. 8:**
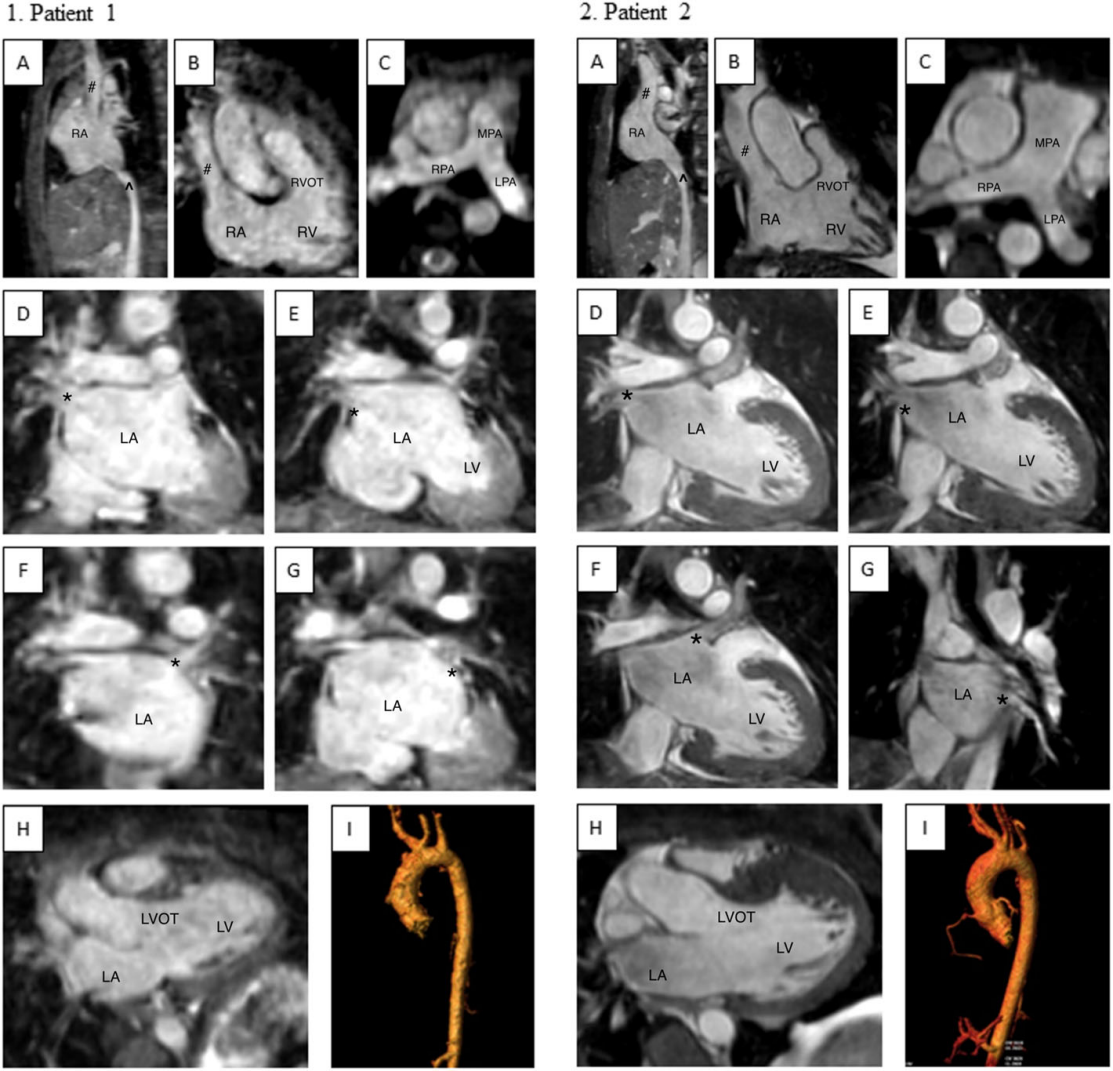
Representative 3D WH bSSFP images from two patients obtained with iNAV. This figure shows the range of image quality that still allowed complete morphological diagnosis (ie. all structures identified without severe blurring [18]) 1. Collection of pictures from one patient with lower quality. Patient presented with chest pain and bicuspid aortic on echocardiogram with a suspected abnormal left coronary artery origin. Complete morphological diagnosis was possible; however, this collection is a representative of patients with slightly lower quality. 1A. SVC and IVC enter into the RA normally. 1B. SVC entering RA normally, RA to RV connection with RVOT visualized. 1C. Normal branch pulmonary arteries. 1D. Right upper pulmonary vein (*) entering into the LA. 1E. Right lower pulmonary vein (*) entering the LA. 1F. Left upper pulmonary vein (*) entering the LA. 1G. Left lower pulmonary vein (*) entering the LA. 1H. LA to LV connection with LVOT visualized. 1I. Ascending aorta, arch, head and neck vessels and descending aorta. The left carotid artery arises from the trunk of the innominate artery. 2. Higher quality pictures from another patient with hypertrophic cardiomyopathy. Complete morphological diagnosis was also possible in this patient. 2A. SVC and IVC enter into the RA normally. 2B. SVC entering RA normally, RA to RV connection with RVOT visualized. 2C. Normal branch pulmonary arteries. 2D. Right upper pulmonary vein (*) entering the LA. 2E. Right lower pulmonary vein (*) entering the LA. 2F. Left upper pulmonary vein (*) entering the LA. 2G. Left lower pulmonary vein (*) entering the LA. 2H. LA to LV connection with LVOT visualized. 2I. Ascending aorta, aortic arch, head and neck vessels and descending aorta. The left carotid artery and innominate artery arise from a common trunk from the arch. There is also a vertebral artery arising directly from the arch. iNAV = image navigator; SVC = superior vena cava (#); IVC = inferior vena cava (^); RA = right atrium; RV = right ventricle; RVOT = right ventricular outflow tract; MPA = main pulmonary artery; RPA = right pulmonary artery; LPA = left pulmonary artery; pulmonary veins labeled with asterisk (*); LA = left atrium; LV = left ventricle; LVOT = left ventricular outflow tract

In assessing cardiac anatomy, iNAV allowed complete morphological diagnosis in 27/30 patients: there were two failures due to an inability in visualizing the right subclavian artery and one failure due to inability to visualize the left upper pulmonary vein. Representative images from two patients are shown in Fig. 8. These findings are similar when assessing cardiac images obtained with dNAV, where there was a complete morphological diagnosis in 27/30 patients. Two failures were secondary to inability to visualize a pulmonary vein (1 right lower pulmonary vein and 1 right upper pulmonary vein) and one failure secondary to inability to visualize the left subclavian artery. There was overlap of one patient whose images obtained with iNAV did not allow visualization of the right subclavian artery while images obtained with dNAV did not show the right upper pulmonary vein.

**Fig. 9:**
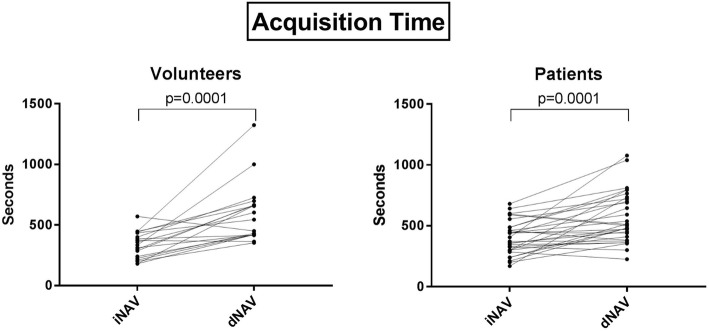
Scan Times of Volunteers and Patients. Acquisition time of 3D WH bSSFP images obtained using iNAV and dNAV for motion correction. This figure shows significantly shorter time for both groups using iNav. iNAV = image navigator; dNAV = diaphragmatic 1D pencil beam navigator

The mean scan time using iNAV was 6:41 ± 2:17 min and 9:57 ± 3:29 min using dNAV, a difference that was statistically significant (*p* = 0.0001). In 24 out of the 30 patients, the scan time was longer using dNAV with the longest scan time using pencil beam lasting 18 min. In contrast, the longest scan time using iNAV was 11:20 min. A comparison of mean scan times for both volunteers and patients between iNAV and dNAV is shown in Fig. 9.

## Discussion

The main finding of this study is that the proposed iNAV motion compensation technique for dual phase 3D WH bSSFP significantly reduces scan time compared to the conventional approach in both healthy subjects and patients with congenital heart disease without significantly compromising image quality. This is likely due to the stricter requirement of the dNAV approach that both the systolic and diastolic navigators be in a narrow 5 mm gating window for each cardiac cycle. This is due to the condition for both systolic and diastolic 3D WH bSSFP acquisition to progress at the same rate, leading to a lower total scan efficiency compared to a similar single-phase 3D WH bSSFP with the dNAV technique. In contrast, the iNAV technique with CRUISE gating uses independent gating windows for systole and diastole, and the nominal scan efficiency of 50% is therefore also the true scan efficiency and the same as a single-phase 3D WH bSSFP scan. A potential drawback of using independent navigators is that the systolic and diastolic CMRA may be slightly misregistered, although in practice that is of minor importance as the two phases are analyzed separately.

Compared to previous studies using dNAV and iNAV for single-phase 3D WH bSSFP [10, 15], there was no improvement in coronary vessel sharpness using iNAV. This is likely related to the stricter gating criteria for the dNAV technique, as previously mentioned. As cardiac cycles only partially inside the gating window (either systolic or diastolic dNAV navigator) are rejected, these acquisitions tend to be on the periphery of the gating window, leading to a narrower distribution of motion values at the center of the gating window. This may subsequently lead to an improvement in image quality due to the narrower range of motion values in the final image. In comparison, for iNAV using independent systolic and diastolic navigators, the gating efficiency is the same for both single and dual phase 3D WH bSSFP and no change in distribution of motion values is expected. As the iNAV approach failed to yield diagnostic image quality in a few cases, imposing stricter gating criteria should be considered. Conversely, the dNAV approach had to be aborted in two patients due to extremely low gating efficiency while a further three cases yielded non-diagnostic image quality. Further work is required to devise a gating strategy which allows dual phase 3D WH bSSFP with high gating efficiency but adaptable to patient breathing pattern or image quality parameters.

Using the proposed technique of independent navigator references, image quality between iNAV and dNAV was similar overall in both healthy volunteers and in patients with congenital heart disease, with a tendency towards being able to image more of the distal vessel with iNAV. The time needed for dual-phase 3D WH bSSFP image acquisition using iNAV, however, was significantly shorter. A complete cardiac morphological diagnosis was possible in the vast majority of cases. In the three cases in which complete morphological diagnosis was not possible, two failures were due to the missing structure being located outside the shim box.

Our findings further illustrate that 3D whole-heart dual phase imaging is adequate for diagnostic ability in patients with congenital heart disease and although both navigation methods (dNAV and iNAV) produce images similar in quality, the time needed to acquire such images is significantly reduced with iNAV. This shortened acquisition time will significantly improve clinical applicability and patient comfort.

In addition to the conventional approach of using a 1-dimensional diaphragmatic navigator (dNAV) and the image-based navigation (iNAV) proposed in our study, there are other strategies to correct for respiratory motion [9]. Another respiratory navigation tool is the so-called “self-navigation”. This navigator, like iNAV, measures respiratory-induced motion of the heart directly which obviates the need for a motion model, however, images are acquired as 1-dimensional projections of the FOV, therefore static structures are also included in the navigator image and may reduce the motion estimation performance of the navigator. Self-navigation was recently evaluated for single-phase 3D WH bSSFP in patients with congenital heart disease [19]. Similar to this study, the self-navigation provided diagnostic image quality in 90% of patients, with a mean scan time of close to 10 min. In this study, a head-to-head comparison between self-navigation and dNAV was performed which primarily demonstrated the value of advanced motion compensation to reduce scan time.

Although in our study, the quality of images obtained with iNAV did not reach a statistically significant difference when comparing against those obtained with dNAV, there were multiple subjects with subjectively improved quality and there was a trend to better distal vessel visualization with iNAV. Further work will investigate the reproducibility of this technique.

A limitation of this study is that iNAV used a different correction and gating approach to dNAV. Therefore, it is not possible to assess the relative contribution of correction or gating when comparing iNAV to dNAV. This is due to a technical limitation, which renders iNAV incompatible with the use of a conventional gating window strategy, and the dNAV navigator is similarly incompatible with CRUISE gating.

As previously mentioned [4, 5], a theoretical limitation of dual-phase imaging is the possible reduction of signal to noise ratio because of the acquisition of two cardiac phases during a single cardiac cycle when compared with the signal-to-noise ratio obtained with a single-phase whole-heart sequence. However, if the scan is performed after contrast administration, as is commonly done, then this signal-to-noise ratio penalty is negligible.

## Conclusions

In conclusion, we have implemented and evaluated a new approach for dual-phase 3D WH bSSFP using image-based navigation. This approach maintains a high accuracy of respiratory motion tracking, despite the different cardiac motion states of the data acquisition, by employing two independent navigator references. It compares favorably to the conventional dNAV approach in both healthy subjects and patients with congenital heart disease, by providing similar vessel sharpness and allowing complete morphological diagnosis for congenital and pediatric heart disease, while significantly reducing scan time.

## Abbreviations

2D: Two-dimensional
3D WH bSSFP: 3D whole heart balanced steady-state free precession
3D: Three-dimensional
AVCD: Atrioventricular canal defect
bSSFP: Balanced steady-state free precession
CRUISE: Constant Respiratory efficiency Using Single End-expiratory threshold
dNAV: Diaphragmatic 1D pencil beam navigator
DORV: Double outlet right ventricle
ECG: Electrocardiographic
FH: Foot-head
FOV: Field of view
iNAV: Image-based navigator
LAD: Left anterior descending artery
LCx: Left circumflex artery
LR: Left-right
MRI: Magnetic resonance imaging
RCA: Right coronary artery
ROI: Region of interest
TGA: Transposition of the great arteries
VSD: Ventricular septal defect

## References

[1] OM W, Martin AJ, Higgins CB (2003). Whole-heart steady-state free precession coronary artery magnetic resonance angiography. Magn Reson Med.

[2] Greil G, Tandon AA, Silva Vieira M, Hussain T (2017). 3D whole heart imaging for congenital heart disease. Front Pediatr.

[3] Sorensen TS, Korperich H, Greil GF (2004). Operator-independent isotropic three-dimensional magnetic resonance imaging for morphology in congenital heart disease: a validation study. Circulation.

[4] Uribe S, Hussain T, Valverde I (2011). Congenital heart disease in children: coronary MR angiography during systole and diastole with dual cardiac phase whole-heart imaging. Radiology.

[5] Hussain T, Lossnitzer D, Bellsham-Revell H (2012). Three-dimensional dual-phase whole-heart MR imaging: clinical implications for congenital heart disease. Radiology.

[6] Brittain JH, Hu BS, Wright GA, Meyer CH, Macovski A, Nishimura DG (1995). Coronary angiography with magnetization-prepared T2 contrast. Magn Reson Med.

[7] McConnell MV, Khasgiwala VC, Savord BJ (1997). Comparison of respiratory suppression methods and navigator locations for MR coronary angiography. AJR Am J Roentgenol.

[8] Uribe S, Tangchaoren T, Parish V (2008). Volumetric cardiac quantification by using 3D dual-phase whole-heart MR imaging. Radiology.

[9] Henningsson M, Botnar RM (2013). Advanced respiratory motion compensation for coronary MR angiography. Sensors (Basel).

[10] Henningsson M, Koken P, Stehning C, Razavi R, Prieto C, Botnar RM (2012). Whole-heart coronary MR angiography with 2D self-navigated image reconstruction. Magn Reson Med.

[11] Henningsson M, Smink J, Razavi R, Botnar RM (2013). Prospective respiratory motion correction for coronary MR angiography using a 2D image navigator. Magn Reson Med.

[12] Scott AD, Keegan J, Firmin DN (2011). Beat-to-beat respiratory motion correction with near 100% efficiency: a quantitative assessment using high-resolution coronary artery imaging. J Magn Reson Imaging.

[13] Wu HH, Gurney PT, Hu BS, Nishimura DG, McConnell MV (2013). Free-breathing multiphase whole-heart coronary MR angiography using image-based navigators and three-dimensional cones imaging. Magn Reson Med.

[14] Henningsson M, Hussain T, Vieira MS (2016). Whole-heart coronary MR angiography using image-based navigation for the detection of coronary anomalies in adult patients with congenital heart disease. J Magn Reson Imaging.

[15] Henningsson M, Smink J, van Ensbergen G, Botnar R (2018). Coronary MR angiography using image-based respiratory motion compensation with inline correction and fixed gating efficiency. Magn Reson Med.

[16] Botnar RM, Stuber M, Danias PG, Kissinger KV, Manning WJ (1999). Improved coronary artery definition with T2-weighted, free-breathing, three-dimensional coronary MRA. Circulation.

[17] Etienne A, Botnar RM, Van Muiswinkel AM, Boesiger P, Manning WJ, Stuber M (2002). “soap-bubble” visualization and quantitative analysis of 3D coronary magnetic resonance angiograms. Magn Reson Med.

[18] Makowski MR, Wiethoff AJ, Uribe S (2011). Congenital heart disease: cardiovascular MR imaging by using an intravascular blood pool contrast agent. Radiology.

[19] Monney P, Piccini D, Rutz T (2015). Single Centre experience of the application of self navigated 3D whole heart cardiovascular magnetic resonance for the assessment of cardiac anatomy in congenital heart disease. J Cardiovasc Magn Reson.

